# Embodied and exbodied mind in clinical psychology. A proposal for a psycho-social interpretation of mental disorders

**DOI:** 10.3389/fpsyg.2015.00236

**Published:** 2015-03-03

**Authors:** Alberto Zatti, Cristina Zarbo

**Affiliations:** ^1^Department of Human and Social Science, University of Bergamo, Bergamo, Italy; ^2^Human Factors and Technology in Healthcare, University of Bergamo, Bergamo, Italy

**Keywords:** embodiment, exbodiment, body, mind, clinical psychology

## Abstract

A brief theoretical review of the current state of the art of embodiment research in clinical psychology has been expounded in order to highlight the key role that embodied conceptualization has on the understanding and explanation of several mental disorders, such as eating disorders, schizophrenia and depression. Evidence has suggested that mental disorders may be explained as disturbances of embodiment, from the disembodiment to the hyperembodiment. In order to understand how some clinical conditions are affected by cultural models, we propose and define a new framework called Exbodiment, complementary to the Embodiment approach to cognition. Mental disorder is strictly related to the subject-culture interaction that may be explained as a two way process in which embodiment and exbodiment are complementary points of view. In this perspective, embodiment may be seen as the “top-down” process, while exbodiment the “bottom-up” one. The introduction of exbodiment conceptualization highlights how subject is both receiver and interpreter of social influence. Subject is the target of a cultural pressure and, at the same time, enacts its own embodied culture in world. Exbodiment conceptualization may help clinicians to better understand and explain the role of culture in the onset and maintenance of mental disorders.

## INTRODUCTION

Psychological processes are influenced by body morphology, sensory and motor systems, and emotions (Glenberg, 2010). Embodiment conceptualization refers to the act of embody, the role that body plays in shaping the mind.

Embodiment is a concept used in many different research areas showing several related meanings. Embodiment has been studied since the early 1970s and 1980s in various domains, like robotics, philosophy, linguistic, neuroscience, and social psychology (Reimann et al., 2012). In every one of these areas, a continuum of theoretical approaches, ranging from strongly embodied to unembodied, exists (Meteyard et al., 2012). Embodiment can be conceptualized as a framework that can provide an unifying perspective for psychological research. This is especially true for the clinical psychology area and the psychopathological one, where embodied cognition theory may allow a better understanding of mental disorder patients (Fuchs, 2009).

Despite the significant role that embodiment perspective takes for understand and explain psychological processes and disorders, some limitations should be taken into account in order to redefine the concept in a new perspective that focuses also on its complementary part, that is exbodiment.

The main aims of this proposal paper is to provide a brief review of the state of the art of embodiment and its application in clinical psychology, to highlight its potential limits and, finally, to introduce exbodiment conceptualization and its promising application in clinical psychology.

## EMBODIED MIND

The conceptualization of Embodiment, or Embodied Cognition, has been introduced between 1970 and 1980 in order to highlight how psychological phenomena are grounded in body, especially in organism’s sensory-motor experiences (Glenberg, 2010; Price et al., 2012; Winkielman et al., 2015), and in social interactions (Fuchs, 2009).

The Embodied Cognition Theory was specifically developed in opposition to the mind–body separation and its latest developments made by cognitivism and computational science (Meteyard et al., 2012). In this field computation was used as a tool to study human mind, and specifically human cognition, adopting a symbol processing approach: mind as a computer, totally detached from body (Meteyard et al., 2012).

Embodied Cognition Theory proposes instead that a functional unity between “low” level sensory-motor and corporeal experiences and cognitive/perceptual processes exists; information processing can accordingly be influenced, modified, or entirely depends on body experiences (Jirak et al., 2010; Winkielman et al., 2015). According to this perspective, cognitions and emotions develop in the sensorimotor experience of the environment (Fuchs and Schlimme, 2009; Price et al., 2012). The interrelation between mind, body, and environment allows subject to shape its particular vision of the world (Costa et al., 2013).

The key-concept of Embodiment is Action, who truly combines mind and body (Fuchs, 2009; Glenberg, 2010; Meteyard et al., 2012; Glenberg et al., 2013). The final target of the human being is *action/interaction* with the world, which is guided by the body; emotive and cognitive processes have their roots on body because they are interlinked and exist to guide action itself (Fuchs, 2009; Glenberg, 2010; Meteyard et al., 2012; Glenberg et al., 2013). Our body movement experiences allow us to recognize how we anticipate events and interact with others (Cipolletta, 2013).

Embodiment biological basis research started in 1990 and focused on the body’s role in shaping and building emotions, conscience and others cognitive aspects of the mind (Varela et al., 1992; Damasio and Dolan, 1999; Fuchs and Schlimme, 2009), contributing to find experimental behavioral and neurological evidence to the Embodiment Theory (Costa et al., 2013). One of the first evidence of Embodied Cognition Theory came from studies on different effects of head movements on cognition (Reimann et al., 2012). Evidence suggests the hypothetical role of mirror neuron system in embodiment processes (Price et al., 2012).

## EMBODIMENT IN CLINICAL PSYCHOLOGY: PSYCHOPATHOLOGY AS BODY DISTURBANCE

Evidence in clinical psychology has suggested that patients with mental disorder embody both their pathology. Applying embodiment in clinical psychology help us to better understand how body features may be taken into account in order to explain mental disorder symptoms (Glenberg, 2010). Psychological and psychopathological disorders may be explained as disturbance of embodiment (Fuchs and Schlimme, 2009). From this point of view, embodiment impairments have been classified in disturbances that primarily affect the subject body or sense of self (e.g., schizophrenia and depression), and disturbances that are more related to the body image and body awareness (e.g., eating disorder; Fuchs and Schlimme, 2009).

Studies that have suggested a major comprehension of three of the main psychopathologies of our century (e.g., eating disorder, schizophrenia, and depression) in relation to embodiment and corporality themes have been briefly reviewed.

### EMBODIMENT IN EATING DISORDER

#### Eating disorder as a body language

Embodiment has been suggested to play a key role in eating disorders because of the strict link between body, culture and identity. Our body, that is cultural, allows us to communicate with others and with ourselves. In the interaction between body and culture, identity and self-esteem play a key role and are closely linked to body image. In this interaction, the external features of my body (such as sizes, weight, muscles) are tools that subjects use in order to show and promote their internal life (Cohane and Pope, 2001). Body is then both physical and symbolic. Body features lead subjects to express psychological and internal meanings and emotions. Body is so a symbolic language that allows subject to concretize emotions and internal states.

A recent study of adolescents in residential care and at risk for eating and related body-image disorders has found that most of the sample has experienced troubled events in their lives, such as loss, abuse, deprivation, and parental neglect (Skarderud et al., 2005). The authors have so theorized that adolescents at risk of eating disorders experience the so-called embodiment of troubled lives. Moreover, most of them explain their own need for control, and the related feeling of chaos when they can not control themselves and events. These features suggest that eating disorder symptoms are bodily messages about patients’ difficulties in controlling life events (Skarderud et al., 2005). The troubled body may be a symbol of a troubled existence that lacks of essential control over life conditions (Skarderud et al., 2005).

#### Eating disorder as an alienation of own body and emotions

Evidence has suggested and demonstrated that patients with eating disorders are affected by disturbances of the way they experience and get value to their own body (Stanghellini et al., 2012). It is well-known that patients with eating disorder overvalue their body shape and weight. This disturbance may be due to a specific disorder of lived corporeality, in particular to a predominance of a specific dimension, that is the “lived-body-for-others.” The “lived-body-for-others” concerns the awareness that my own body is looked by other subjects and that it is an object for them. This awareness allows me to experience the revelation of “my being as object,” affecting then my own identity. As a result, eating disorder patients are alienated from their own body and emotions, and experience body as an object that is looked at and judged by other subjects (Stanghellini et al., 2012).

#### Eating disorder as disembodiment expression

Bodily impairments, common in anorexia nervosa, allowed scientific community to think that these patients experience impairments in body awareness and contact. Anorexia nervosa has been then proposed as a disorder of embodiment, a disembodiment, that imply a disconnection with own body (Kolnes, 2012). Patients with anorexia nervosa tend to dissociate head from body.

Evidence has shown that patients with anorexia nervosa have significant impairments in their body awareness, muscular tension, restricted breathing pattern, and postural disturbances (Kolnes, 2012). Moreover patients show inability to relax and a compulsive physical activity style (Kolnes, 2012). In addition, they show confusion concerning bodily sensations and states, and inability to describe bodily experiences. Sometimes they report having not a body, and that they exist only as a subject in their head. Common is the inability to perceive, understand and respond to bodily signals, in particular concerning visceral sensations related to hunger (Bruch, 1962, 1988; Pollatos et al., 2008; Thornborg and Mattson, 2010). In addition, eating disorder patients show specific personality traits (Melchionda et al., 2003; Dalle Grave et al., 2013) and lower mindfulness capacities, if compared with controls (Compare et al., 2012).

### EMBODIMENT IN DEPRESSION

#### Depression as hyperembodiment

Depressive disorder has been conceptualized as an hyperembodiment expression. In depressive patients body becomes conspicuous, heavy and solid. The body of a depressed subject puts up resistance to the subject’s intentions and impulses (Fuchs and Schlimme, 2009). Body is experienced as heavy, oppressive, rigid and as an impairment to everyday normal life. Depressed subject is not capable to embody phenomenal space and identify himself with its own body. He focuses on bodily failure and, as a results, he feels worthless, guilty and decaying (Fuchs and Schlimme, 2009). In depressive disorder and melancholia, the self is disconnected by the body and the sense of being alive is lost.

#### Depression and body systems

Studies on depressed patients have moreover demonstrated a strict link between motor systems and emotional processes (Sloman et al., 1982; Michalak et al., 2009, 2014). It has been suggested that bodily manipulations may affect emotionally negative and positive stimuli processing. For example, bodily movements such as pushing a lever away from their body rather than pulling it toward it, affect profoundly the emotionally processing of positive or negative stimuli (Duckworth et al., 2002).

Mood states have direct effects on the way people walk. Then, said patients walk with a lifting motion of the leg, whereas normal control subjects propel themselves forward (Sloman et al., 1982). Recently, a study has demonstrated the existence of a specific gait pattern in depressed and said subjects; they are characterized by reduced walking speed, arm swing, vertical movement of the head, stronger lateral body sway, and a slumped posture. In this pattern, a key role play speed and posture (Michalak et al., 2009).

Studies on embodiment in depressive disorder, have found that embodied experiences have direct effects also on memory recall (Dijkstra et al., 2007; Michalak et al., 2014; Winkielman et al., 2015). In turn, negative memory recall and rumination have been linked to the exacerbation and maintenance of depressed mood (Compare et al., 2014).

Evidence shows that depressed patients that sit in a slumped posture, during a laboratorial experiment of recalling, refer more negative than positive words if compared with patients that sit in upright posture; depressed patients in an upright posture show, in fact, a balanced recall of positive and negative words (Michalak et al., 2014). Moreover, it has been suggested that depressed patients retrieve more easily and in shorter time memories when their posture is congruent with the mood relevant to the memory, rather than incongruent (Dijkstra et al., 2007). Posture has significant effects on power perception. Posture has been, in fact, associated with the production of high or low social power behaviors (Huang et al., 2011).

### EMBODIMENT AND SCHIZOPHRENIA

#### Schizophrenia as disembodiment expression

Recent phenomenological approaches suggest that schizophrenia is a disorder of embodiment, that is a disturbance of the embodied of self (Fuchs and Schlimme, 2009; Stanghellini, 2009). The weak sense of self, the break of bodily functioning and the disconnection of intercorporeality with others, lead to a loss of the link between self and world. The disembodiments of the self, of the self-object relation and of interpersonal relationships lead patients to live and act as a soulless body or a disembodied spirit. Schizophrenic patient experiences a sense of mechanization of the body, feeling like a “psycho-machine” (Stanghellini, 2009).

In schizophrenia, personal and social spaces are affected. Schizophrenic patients make significant errors in determining boundaries of peripersonal space when interacting both with an object and with a subject. This inability leads patients to show impairment in social integration because they are unable to adapt to social and common norms (Delevoye-Turrell et al., 2011).

Schizophrenia adversely affects also how patients embody actual states and hopes for future (McCann and Clark, 2004). Schizophrenia patients experience frightening, embarrassment and sense of guilt for something that they have done or said. Common is the lack of confidence in themselves, social isolation and the reluctance to trust others.

The loss of their own body is strictly related to the loss of their own mind. Patient loses his self because his body no longer acts in the way that is familiar and trust for himself. These sensations may increase if medications affect its own appearance (McCann and Clark, 2004).

The embodiment of temporality, relationality and treatment are significant themes for schizophrenic patients (McCann and Clark, 2004). In particular, studies have shown that schizophrenia is experienced as a catastrophic event that affects deeply patients’ temporality and lived time, because of delusions and hallucinations (Embodied temporality; McCann and Clark, 2004). Illness is also experienced as a mediator of social relationships that affects their quality. Schizophrenia has a paradoxical effect on social relationships, because of sometimes it helps at elicit support, while sometimes damage social relationships (Embodied relationality; McCann and Clark, 2004). Also antipsychotic medications affect patients’ perception of their own body images and impair the ability to have sexual and social relationships (Embodied treatment; McCann and Clark, 2004).

Schizophrenia affects perception of objects, selves, and bodily functioning (Fuchs and Schlimme, 2009).

Typical in schizophrenic patients is the impairment of the ability to recognize familiar patterns of perceived objects, because of the tendency to focus on details and the inability to grasp the overall meaning of things (Fuchs and Schlimme, 2009). This mechanism is maintained by the fact that if a body’s involvement in the world is switched off, then the grasp of the things will be impaired (Stanghellini, 2009). As a result, objects loss the so-called ready-to-hand meanings that would help patients to give practical means to the things of the world (Stanghellini, 2009).

Concerning the perception of the self, the alienation from their own body allows patients to perceive themselves as external spectator of their own perceptions, actions, and thoughts (Fuchs and Schlimme, 2009; Stanghellini, 2009). They frequently experience a feeling of loss of presence that involves the detachment from themselves, their actions and experiences and, in the severest cases, a sense of emptiness (Stanghellini, 2009). The alienation of bodily functioning leads patients to experience a disintegration of daily live habits or automatic practices (Fuchs and Schlimme, 2009).

All of these elements are then organized in delusions that give a new and rigid coherence and meaning to the environment (Fuchs and Schlimme, 2009).

## EMBODIMENT’S LIMITS

As stated before, the concept of Embodiment is frequently used in the basic and “simple” meaning of “mind and body reflect on each other.” In this perspective, depressed subjects walk heavily, schizophrenic patients act and think as in a mechanic body, and anorexic girls perceive their own body fat also if it is underweight.

The embodied “revolution” concerns that somatic-cognitive processes are carried out continuously in the here and now of the intervention on environment. Theorists of enaction use the term “simulation” to indicate the need to reverse the direction in which senses are studied. We should start from the objective body and understand how the brain receptors queries by adjusting the sensitivity, combining posts, pre specifying the estimated values according to an internal simulation of the expected consequences of the action (Berthoz, 1998). The perception is action and action influences the relevance and the cognitive load that is bound to an object.

Action simulation is then directed toward some possible lines of movement and not to all of potential actions. The notion that philosophy uses for this “problem” is “intention.” Intentionality selects data that seems most relevant for action. Actions on things of the world and the interaction between people can be analyzed in terms of “intentional carriers,” that may be explained as guides for behaviors. Interaction between subjects, that is the relational dimension, can also be seen as an over-ordered “channel” where people take out preferences for actions. These intentional meta-subjective goals are called “cultural intentions” because the social group processes them for its members.

Subjects should be considered as intentional human beings. While acting, they project their goals into the world. When these goals diverge from the intentional plan, they intervene to actions in order to correct them. But, at the same time, cultural patterns influence individual behavior, soliciting them to reach a specific typology of social and personal identity.

The “primacy” of the simulation, as stated by Berthoz (1998), also reveals how human subject is not a simple detector of objective data. Imagination is in fact a pillow-like filter between the world and the subject. Perception is not just correct or not. The world shows different things as the perceiver faces it with different intentions. In analogic terms, imagination and simulation are complementary processes with which people exchanges symbols founded in cultural environment. We call this “space” Peripersonal Symbolic Sphere, that is the participative area in which action with objects and interaction with similar is possible in a structured space where cultural norms manifests possibility or prohibitions.

## EXBODIMENT: THE ENACTION OF THE EMBODIED CULTURE

Embodied Cognition and Enaction Theory (Varela et al., 1992) are theoretical perspectives that share the same background. Interaction between subjects and cultures can be then explained as a two way process: embodiment and exbodiment. Oxford Dictionary states: “Embodiment (noun): a tangible or visible form of an idea, quality, or feeling,” as in “she seemed to be a living embodiment of vitality.” Embodiment concerns a “top-down” process and people are conceived as targets of cultural pressure, that is formed of social representations or “ideas” (Farr and Moscovici, 1989). As in Perception Theory, we state here a “top-down process of social models” because subject directs his/her behavior on the basis of cultural solicitations that he/she had encountered. Our social–psychological perspective assumes here the “collective-mind” point of view (Mead, 1934).

We suggest that the interaction between subject and culture is a two way process. We call “exbodiment” the “bottom-up” process. Exbodiment indicates how subjects “enact” their embodied cultures, how the body express ideas, which are never as similar as to the ideal type of a society. This occurs in Peripersonal Symbolic Sphere, where culture is seen as the framework from which (top-down) norms are conceived by people as continuous strength to cope with the influence (Lakoff and Johnson, 1982).

The verb “to enact” comes from philosophy of law language. It underlines the specific ontology of a bill or a legal proposal, because the statue of legal sentences are social facts with a certain grade of objectivity. Action is a more general word, when enaction stresses the institutional nature of the people’s activity. We can not just say that “one acts,” but we propose to say that an oriented enactions, acted by subjects and structured by a culture, construct his/her own social world.

Exbodiment indicates how subjects “enact” their embodied cultures. For example, exbodiment refers to the process applied by anorexic girls to organize the social-echo-system in order to control their attempt to embody the “fitting form” detected in cultural symbols. Embodiment concerns the incoming shape from a culture to a subject, while exbodiment refers to the never ending process by which subject expresses his adhesion to cultural and social model.

### EXBODIMENT IN CLINICAL PSYCHOLOGY

Many psycho-pathological conditions, in particular eating disorders, are sensitive to the social influence. Many clinicians refer to a “social epidemic” event for anorexia showing how factors related to disease can be found also in the socio-cultural dimension of the phenomenon. Also depressive syndromes represent a growing trend in today’s society. Social psychology and cultural anthropology are therefore called to develop explanatory models of these diseases.

Social influence is a complex phenomenon that shapes imagery and “simulation plans” that subjects develop in their mind. In this perspective, subject might not be considered only as a target of social influence. Subject is a speaker, an amplifier and an “interpreter” of its “embodied simulation,” that is often unconscious. Anorexic and bulimic patients are not just passive subjects. They in fact organize their environment and use all of their relational power to influence it.

The “relational effects” of a disease are considered as complex factors that modulate the expression of behaviors, such as anorexic weight loss. Subjects explore (enact) their social environment in order to test the possibilities to conform it to his own pathological and distorted assumptions. As stated by Jean Piaget, the knowledge reaches the object only through the transformation brought by an action and knowledge is structurally related to the action (Ceruti, 1993). The world is not something that is given to us, but is something in which we take part through the way by which we move (Varela et al., 1992). In recent decades, people’s Life Space (Lewin, 1964) has increased in complexity and extensivity, because the communicative sphere of contemporary society includes visual media, social networks and “covered myths” that underlie a given society.

Individual and complex societies are in an unbalanced relationship, because an individual has little power to “change” a state of society that is implemented by powerful agencies and in places far away from their possibility to intervene. Fashion houses that parade manuequine in world’s capitals are not affected by any action of a 15 year old girl. What is out of subject action space is reached by people imagination.

Peripersonal Symbolic Sphere is wider than interactive peripersonal space. Claiming that social communication must bann the lean bodies because they could push thousands of girls to lose weight uncontrollably does not make much sense, both because of its impracticality, and because it goes to affect creative freedom. Our suggestion is that it is possible to answer to the eating disorder social epidemic alarm not just preventing or prohibiting the exposure to the infection stimuli (i.e., the thinning vip of the moment, as a medical optic tend to suggest) but reinforcing the subjective initiative of the Peripersonal Symbolic Sphere.

## CONCLUSION

This proposal paper has provided a brief review of the state of the art of embodiment and its application in clinical psychology, in particular in the understanding of the onset and maintenance of eating disorders, depression, and schizophrenia. Limits of Embodiment perspective have been then discussed in order to introduce the conceptualization of a new complementary perspective, called Exbodiment. Promising applications of Exbodiment in clinical psychology, in particular in the explanation of anorexia, have been then discussed.

Eating disorder, schizophrenia and depression are the most common mental disorders of our century that directly and indirectly affect also our health. For example, depression is a primary risk factor for cardiac disease, influencing heart through biological and behavioral mechanisms (Maier et al., 2007; Compare et al., 2011a,b, 2013). Also eating disorders, in particular obesity, is a complex disease where somatic and psychological/psychiatric factors contribute to severe distress and poor health-related quality of life (HRQL; Petroni et al., 2007).

The introduction of exbodiment conceptualization highlights how subject is both receiver and interpreter of social influence. Eating disorder should be considered not only as a result of influences spread by social imaginary, but also as a way to be “cool” for girls. According to this perspective, control behavior over food, typically performed by anorexic girls, can be explained as a manifestation of the exbodied theme of girl’s life: the incorporation of an aesthetic influencing model.

Enaction theory suggests that changes in personal beliefs depend on actions that a subject makes in his environment. Enacting epistemology remembers that for a living system, the meaning of interactions is not required from the outside, but is the result of the organization and history of the system (Varela et al., 1992). Subject learns by acting, even if in presence of perceptual “distortions,” as in the case of dismorphophobic perception. In this perspective, it has been suggested that cognitive dissonance strategies (Festinger, 1962) may change subjects’ attitudes against beliefs (Stice and Presnell, 2011).

Social passivity may therefore be considered one of the most maintaining cause of mental disorder in our contemporary societies. The more a subject is sustained to be active in the dialectic between embodiment and exbodiment, the greater will be the dynamics of personal identity which could help people to find their place in the society. Because of inequality in influencing power between symbolic agencies, as media-systems and common people, cultural symbols and images could crush personal life. Then, in order to go out of the so called “social emergency” for eating disorders and depressive syndromes, it is necessary to sustain subjective initiative in all educational contexts (better if in in-group situations). Empowering the enactive side of the Peripersonal Symbolic Sphere could help girls to be critical evaluators of social models.

**FIGURE 1:**
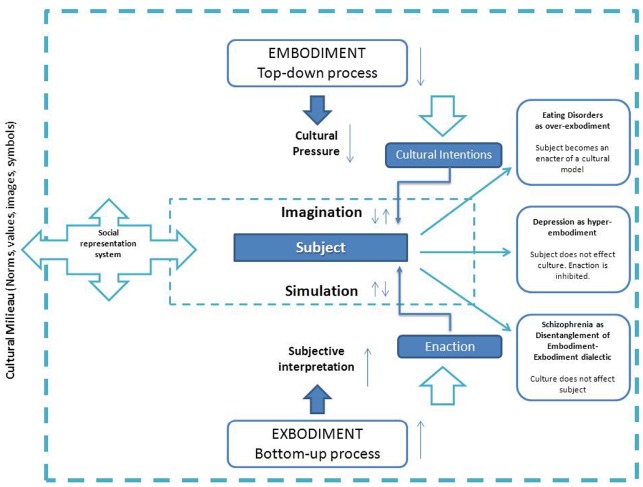
**Embodiment–exbodiment in cultural dialectic model**.

Figure 1 shows as different “accentuations” or “dominances” of one’s embodiment–exbodiment dialectic may help to better understand subjective coping strategies to cultural influences. Cultural milieu is the social representation incubator in which subject receives identity models that are embodied in his individuality. Subject replies to social models through his own enaction modality (exbodiment). The interface between “individual mind” and “collective mind” is constituted by the simulation–imagination system (representation system). Potential results of embodiment–exbodiment interaction are represented in the right side of the figure. Psychological disorders have been reconsidered taking into account their effects on social-relational environment (micro-culture). From the coping effect point of view, eating disorders show an over-exbodiment to cultural model, while depressive disorders show action inhibition. Schizophrenic condition may be characterized as an original disarticulation of social influences and subjective coping link.

A two-way consideration of social diseases would allow clinical psychology to understand how pathological behaviors are result of a complex interaction between cultural, individual and relational factors. In our point of view, clinical psychology should consider that “healthy” actions should not be limited to states defined by diagnostic manuals. The conditions set by psychiatry are “stabilized organizations” in the exbodiment–embodiment relationship. Clinical psychology, like other areas of applied psychology, should therefore adopt appropriate psychosocial categories that allow it to extend its point-of-view even at sub-clinical conditions. In this perspective we have tried to illustrate how social and community psychology could support clinical psychology to better understand mental disorders, adopting suggestions from enaction and embodiment–exbodiment theories.

### Conflict of Interest Statement

The authors declare that the research was conducted in the absence of any commercial or financial relationships that could be construed as a potential conflict of interest.
